# Asymptomatic *Yersinia pestis* Infection, China

**DOI:** 10.3201/eid1109.041147

**Published:** 2005-09

**Authors:** Min Li, Yajun Song, Bei Li, Zuyun Wang, Ronghai Yang, Lingxiao Jiang, Ruifu Yang

**Affiliations:** * Qinghai Institute for Endemic Diseases Prevention and Control, Xining, People's Republic of China;; † Beijing Institute of Microbiology and Epidemiology, Beijing, People's Republic of China

**Keywords:** Plague, Yersinia pestis, serodiagnosis, hemagglutination, Western blotting, protein microarrays, letter

**To the Editor:** Plague is one of the oldest identifiable diseases. Modern public health measures and effective antimicrobial treatments have led to a decrease in plague cases worldwide. However, plague remains endemic in many natural foci. Since the early 1990s, the World Health Organization (WHO) has reported a steadily increasing trend in human plague cases, which has resulted in the recognition of plague as a reemerging disease (*1*). The emergence of antimicrobial drug–resistant strains of *Yersinia pestis*, along with an increasing number of plague cases, remind us that plague still poses a serious public health threat (*2**,**3*). In China, human cases of plague have been reported to WHO nearly every year from 1989 to 2003; these account for 9.5% of cases and 15.5% of deaths from this disease in Asia (*1*). Human cases of plague in China are usually caused by contact with plague-infected rodents. Here, we report the results of a serologic survey by using 3 methods (passive hemagglutination assay, Western blot, and protein microarray analysis) in marmot hunters in Qinghai Province, China.

One hundred twenty serum samples were collected in 2 villages in Huangyuan County, Qinghai Province, from marmot hunters (63 samples) and their family members (57 samples); none had a history of fever in the past 2 years. One hundred nineteen serum samples were collected from persons with no history of marmot hunting in 2 nearby counties in Qinghai Province in which plague was not endemic. Thirty serum samples were collected from persons in Beijing and used as negative controls.

All serum samples were initially screened with a passive hemagglutination assay to detect immunoglobulin (Ig) G antibody against F1 antigen of *Y. pestis*, by using a standard protocol (*4*). We then used an F1 antigen–based Western blot to analyze all serum samples. The protein microarray analysis was performed with 149 purified recombinant proteins of *Y. pestis* (*5*).

The results of the serologic survey are summarized in the Table. The passive hemagglutination assay showed 17 positive samples in the marmot hunter population. None of the control serum samples were positive for F1 antigen in this assay. Western blot identified 9 additional positive samples in the marmot hunter population, resulting in a seropositivity rate of 21.7% (26/120). We also found positive samples in 4 (3.4%) of 119 serum samples by using Western blot in persons from areas in which plague was not endemic. Identical results were also obtained by using protein microarray analysis, which validated the results of Western blot.

**Table:** Analysis of sera for plague antibody by 3 methods

Method*	Marmot hunter population, no. positive/no. tested (%)			Population from nonendemic areas, no. positive/no. tested (%)		
	Male	Female	Total	Male	Female	Total
PHA	16/68 (23.5)	1/52 (1.9)	17/120 (14.2)	0/60 (0)	0/59 (0)	0/119 (0)
WB	25/68 (36.8)	1/52 (1.9)	26/120 (21.7)	3/60 (5.0)	1/59 (1.7)	4/119 (3.4)
PMA	25/68 (36.8)	1/52 (1.9)	26/120 (21.7)	3/60 (5.0)	1/59 (1.7)	4/119 (3.4)

*PHA, passive hemagglutination assay; WB, Western blot; PMA, protein microarray analysis.

Previous studies have shown that plague antibodies were more prevalent in males in the exposed population, and differences in the age, sex, or ethnic group of plague patients are the result of variations in exposure to the pathogen, not intrinsic factors (*6**,**7*). Our study showed that in the marmot hunter population, the plague seropositivity rate was significantly higher in males (36.8%, 25/68) than in females (2.0%, 1/52, p<0.01). Among the marmot hunter population, 63 (92.6%) of 68 males were hunters. Plague antibodies were also more prevalent in marmot hunters (39.7%, 25/63) than in their family members (1.8%, 1/57, p<0.01).

This is the first serologic survey of plague in the marmot hunter population. The plague seropositivity rate of 21.7% (26/120) in hunters and their families is much higher than the 3.4% (4/119) in the population from regions in which plague was not endemic (p<0.01). Seroprevalence in marmot hunters was even higher (39.7%), which suggests that marmot hunting is a risk factor for plague infection.

The marmot (*Marmota himalayana*) is the main host of *Y. pestis* in Qinghai Province. Plague-infected marmots are more easily captured by hunters. When persons hunt and butcher marmots without any effective protection, *Y. pestis* can be transmitted through tiny wounds in the skin, by bites of infected fleas, or by the respiratory route. Asymptomatic plague infection in marmot hunters might be explained by prophylactic use of antimicrobial drugs. Most hunters usually take sulfamethoxazole or tetracycline as a prophylactic measure. Even if the hunters were infected with *Y. pestis*, they would likely not develop symptomatic plague. However, if the antimicrobial drugs are not effective or hunters do not use prophylaxis, symptomatic infections will occur. Most reported human cases of plague in Qinghai Province were caused by hunting or butchering marmots, as shown by a recent outbreak of plague in October 2004 in Qinghai, in which 19 cases were reported and 8 persons died (M. Li et al., unpub. data).
